# Novel Bunyavirus in Domestic and Captive Farmed Animals, Minnesota, USA

**DOI:** 10.3201/eid1909.130165

**Published:** 2013-09

**Authors:** Zheng Xing, Jeremy Schefers, Marc Schwabenlander, Yongjun Jiao, Mifang Liang, Xian Qi, Chuan Li, Sagar Goyal, Carol J. Cardona, Xiaodong Wu, Zerui Zhang, Dexin Li, James Collins, Michael P. Murtaugh

**Affiliations:** College of Veterinary Medicine, University of Minnesota—Twin Cities, St. Paul, Minnesota, USA. (Z. Xing, J. Schefers, M. Schwabenlander, S. Goyal, C.J. Cardona, J. Collins, M.P. Murtaugh);; Jiangsu Provincial Center for Disease Prevention and Control, Nanjing, China (Y. Jiao, X. Qi);; Chinese Center for Disease Control and Prevention, Beijing, China (M. Liang, C. Li, D. Li);; Medical School and State Key Laboratory of Pharmaceutical Biotechnology–Nanjing University, Nanjing (Z. Xing, X. Wu, Z. Zhang)

**Keywords:** bunyavirus, phlebovirus, heartland virus, severe fever with thrombocytopenia syndrome virus, animals, infection, viruses

## Abstract

We tested blood samples from domestic and captive farmed animals in Minnesota, USA, to determine exposure to severe fever with thrombocytopenia syndrome virus and Heartland-like virus. We found antibodies against virus nucleoproteins in 10%–18% of samples from cattle, sheep, goats, deer, and elk in 24 Minnesota counties.

Severe fever with thrombocytopenia syndrome (SFTS) is an emerging infectious disease in China, caused by a novel bunyavirus in the genus *Phlebovirus* (*1*). As many as 10,000 SFTS case-patients have been reported since disease emergence in 2009, with fatality rates ranging from 2% to 15% (*1*,*2*), mainly in the eastern provinces of China. SFTS bunyavirus (STFSV) appears to be transmitted by ticks, an unusual difference from other pathogenic phleboviruses, which are transmitted primarily by mosquitoes (*3*). Recently, a new phlebovirus, named the Heartland virus (HLV), was isolated in Missouri from patients with a history of tick bites and signs and symptoms similar to those of SFTS, including high fever and low blood leukocyte and platelet counts (*4*). Phylogenetic analysis showed that HLV is closely related to SFTSV, which suggests that this new phlebovirus could be a serious threat to public health in the United States.

Many bunyaviruses can infect animals (*3*). Little is known about the animal host species that carry HLV or HLV-like bunyaviruses in the United States. Serologic surveys in China found that farm animals, including cattle, goats, and sheep, were infected with SFTSV in disease-endemic areas. In these studies, viral RNA was identified in animal serum specimens, and these isolates shared high sequence homology with isolates from humans (*5*). Strikingly, up to 47% of farm animals in Jiangsu Province, China, had SFTSVs (*4*), indicating that active virus transmission is occurring in the rapidly expanding disease-endemic area. It is critical to identify animal hosts that may be susceptible to, and infected with, HLV or an SFTSV-like virus, and may serve as amplifying hosts that facilitate virus transmission in the United States. To identify animal hosts that may play an essential role in transmission of SFTSV- or HLV-like viruses in the United States, we conducted serologic testing of samples collected from farm animals in Minnesota, USA. Our findings raise the specter of widespread distribution of a novel pathogen among livestock and wildlife that has the potential to be transmitted to humans. 

## The Study

Blood samples, obtained from several domestic and captive farmed animals of various species, were analyzed at the Minnesota Veterinary Diagnostic Laboratory, College of Veterinary Medicine, University of Minnesota. Samples had been collected from September 8 through October 12, 2012, from cattle, goats, sheep, and elk and white-tailed deer andwere submitted mainly for routine surveillance purposes from 29 Minnesota counties.

No HLV or SFTSV antibody test kits are currently available in the United States. We found that anti-SFTSV nucleoprotein (NP) antibodies cross-react with HLV NP and decided to use SFTSV NP antibody detection kits for detecting antibodies against SFTSV- or HLV-like viruses. A standard ELISA reagent kit, developed by Jiangsu Centers for Disease Control (*6*), was used to detect all subtype antibodies specific to the SFTSV NP, following the providers’ instructions. Both positive and negative controls were included, and the results of an assay were considered acceptable when the optical density (OD) of the positive and negative controls were ≥1.50 and ≤0.10, respectively. Samples with an OD value ≥2.1× the mean negative control were considered positive (*6*). Positivity/negativity ratios were calculated for all samples tested, and N represents the mean OD value of negative controls.

**Figure 1:**
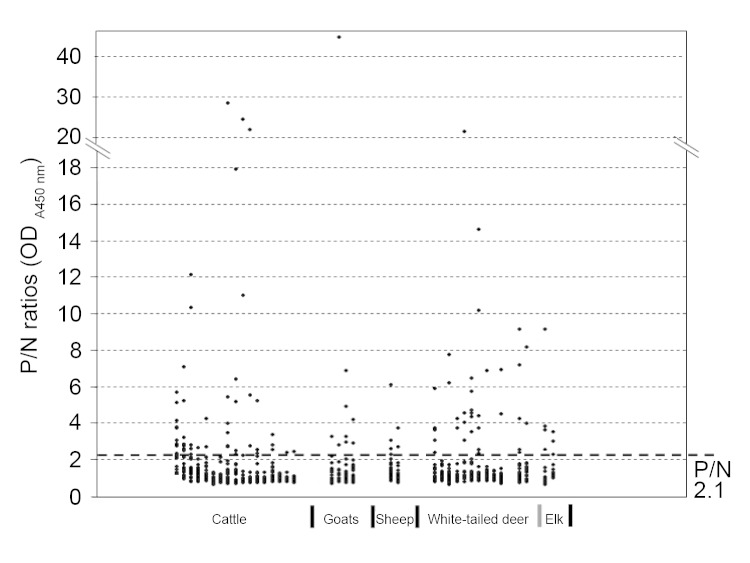
Distribution of positivity (P)/negativity (N) ratios among various animal species tested for antibodies against severe fever with thrombocytopenia syndrome virus nucleoprotein, Minnesota, USA, 2012. N = mean + 3 × SD of optical density (OD)_450nm_ values of negative controls; P = OD_450nm_ value of a test sample.

**Figure 2:**
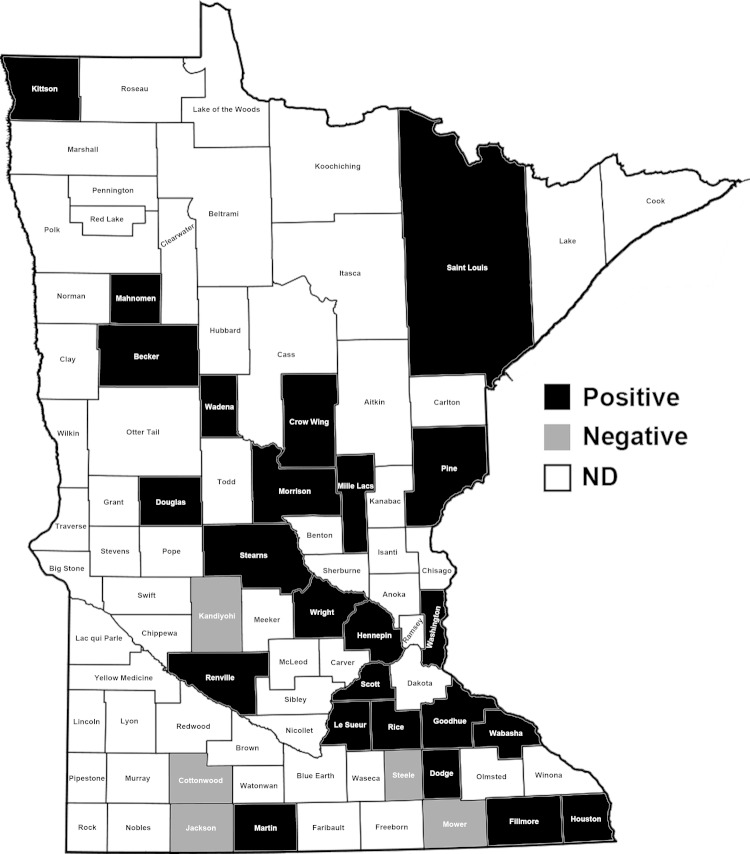
State of Minnesota showing counties. Domestic and captive farmed animals positive for antibodies against severe fever with thrombocytopenia syndrome virus nucleoprotein were found in 24 (black) of 29 counties, 2012.

Antibodies were detected in serum samples from 64 (15.5%) of 414 cattle, 10 (10.9%) of 92 goats, 6 (12.5%) of 48 sheep, 35 (11.8%) of 296 white-tailed deer, and 7 (18.0%) of 39 elk (17.9%) (Table). Thirty-four of 64 positive samples had positivity/negativity ratios of 4–10, and 11 had ratios >10 (Figure 1). Specific antibody titers of these 11 samples ranged from 80 to 1,280 as determined by serial titration. The positive samples came from 24 of 29 counties tested (Figure 2).

**Table:** Prevalence rates of samples positive for antibodies against SFTSV NP, Minnesota, USA, 2012*

Species	No. tested	No. (%) positive†
Cattle	414	64 (15.5)
Goat	92	10 (10.9)
Sheep	48	6 (12.5)
White-tailed deer	296	35 (11.8)
Elk	39	7 (18.0)
Total	889	122 (13.7)

*SFTSV, severe fever with thrombocytopenia syndrome virus; NP, nucleoprotein; samples were tested by ELISA. †A sample with a positive/negative ratio ≥2.1 was considered positive.

## Conclusions

Our data show that both domestic and captive farmed animals in Minnesota were exposed to SFTSV- or HLV-like virus, as evidenced by the presence of antibodies reactive against the NP of SFTSV at a prevalence ranging from 10% to 18%. Because SFTSV and HLV are closely related (*4*), the viruses detected in this region are most likely HLV or close relatives of HLV. Although NPs of SFTSV and HLV are antigenically cross-reactive, the observed detection rates may be underestimated because the reagents were developed and optimized for SFTSV NP antibodies. A more specific serologic test targeting HLV-specific antigens is under development; it is expected to more accurately assess the prevalence of HLV- or HLV-like virus among animals and humans.

Many arboviruses, including bunyaviruses, are zoonotic pathogens. A high fatality rate has been reported for sheep, goats, cattle, and wildlife infected with Rift Valley fever virus (RVFV) (*7*,*8*), and infections have also led to abortion in 100% of pregnant livestock. Whether the SFTSV- or HLV-like virus detected in this study is pathogenic to animals and can cause disease if transmitted to humans remains to be determined. Our records have shown that all animals that tested positive did not exhibit apparent clinical signs even though infected. Because most samples, including almost all seropositive ones, were sent to the Minnesota Veterinary Diagnostic Laboratory for surveillance purpose, the time of the animal’s infection with HLV-like virus is uncertain, but the animals were apparently healthy when sampled. However, we cannot exclude the fact that the animals may have shown clinical signs when infected with the virus.

Vertebrate animals are amplifying hosts for many arboviruses, which have seasonal epidemics (*3*). Distinct strategies are used by various arboviruses for interepidemic virus maintenance. Bunyaviruses, except for hantaviruses, are obligate vector-borne viruses, and their vectors include mosquitoes, ticks, and sandflies. La Crosse virus of the genus *Orthobunyavirus *is transmitted by mosquitoes, and rodents and foxes can be infected as amplifying hosts during seasonal outbreaks (*9*). RVFV is transmitted by mosquitoes as well and can infect a variety of livestock including cattle, goats, and sheep, in which the virus is amplified and transmitted to humans who are in close contact with viremic animals (*10*). Although a bite from an infected mosquito is critical for human infection, humans are more likely to become infected with RVFV through direct contact with viremic animals especially during the process of animal birth or abortion (*11*). RVFV is also confirmed to be highly infectious in aerosols (*12*).

SFTS epidemics in China are seasonal and occur from late March through early November. Infection rates among humans and animals rise in early March, peak in August, and decrease after November (*1*,*2*). The coincidental pattern of human epidemics and animal infections indicates that infected livestock play a critical role as amplifying hosts in SFTS epidemics. In Minnesota, the SFTSV- or HLV-like virus infects a variety of ungulates, both domesticated and wild. We also show that deer and elk (cervids) may be susceptible to this virus in this region. Farmers, hunters, and persons with outdoor lifestyles may become infected when they are bitten by infected ticks. In addition, direct contact with secretions, body liquids, or feces from viremic animals would also put these persons and veterinarians at risk, if HLV- infected animals have substantial amounts of virus in blood and other tissues. The direct contact transmission of SFTSV has been reported in family clusters among persons with no history of tick bites, suggesting that person-to-person transmission may also occur (*13**–**15*).

Evidence that a novel phlebovirus infects domesticated and captive farmed animals as shown in this study validates the concern that an SFTSV- or HLV-like emerging pathogen could pose a serious public health threat in the United States. Epidemiologic studies with a broader scope need to be conducted to elucidate viral ecology, and effective measures must be adopted to control this virus before it spreads among humans.
